# Root hairs aid soil penetration by anchoring the root surface to pore walls

**DOI:** 10.1093/jxb/erv560

**Published:** 2016-01-21

**Authors:** A. Glyn Bengough, Kenneth Loades, Blair M. McKenzie

**Affiliations:** ^1^The James Hutton Institute, Invergowrie, Dundee DD2 5DA, UK; ^2^School of Science and Engineering, University of Dundee, Dundee DD1 4HN, UK

**Keywords:** Biopores, root growth, root:soil contact, root systems, *Zea mays*.

## Abstract

The physical role of root hairs in anchoring the root tip during soil penetration was examined. Experiments using a hairless maize mutant (*Zea mays*: *rth3–3*) and its wild-type counterpart measured the anchorage force between the primary root of maize and the soil to determine whether root hairs enabled seedling roots in artificial biopores to penetrate sandy loam soil (dry bulk density 1.0–1.5g cm^−3^). Time-lapse imaging was used to analyse root and seedling displacements in soil adjacent to a transparent Perspex interface. Peak anchorage forces were up to five times greater (2.5N cf. 0.5N) for wild-type roots than for hairless mutants in 1.2g cm^−3^ soil. Root hair anchorage enabled better soil penetration for 1.0 or 1.2g cm^−3^ soil, but there was no significant advantage of root hairs in the densest soil (1.5g cm^−3^). The anchorage force was insufficient to allow root penetration of the denser soil, probably because of less root hair penetration into pore walls and, consequently, poorer adhesion between the root hairs and the pore walls. Hairless seedlings took 33h to anchor themselves compared with 16h for wild-type roots in 1.2g cm^−3^ soil. Caryopses were often pushed several millimetres out of the soil before the roots became anchored and hairless roots often never became anchored securely.The physical role of root hairs in anchoring the root tip may be important in loose seed beds above more compact soil layers and may also assist root tips to emerge from biopores and penetrate the bulk soil.

## Highlight

Root hairs anchored maize roots to the sides of pore walls, helping root tips to penetrate soil at intermediate soil bulk densities, shown by comparing wild-type seedlings with hairless mutants.

## Introduction

Plant growth and crop yield depend on plants acquiring adequate nutrients and water from the soil. This requires a root system that is in close contact with a sufficiently large volume of soil to deliver these water and nutrients. Root growth in arable soils is often limited by soil strength (Bengough *et al.*, 2011; Valentine *et al.*, 2012), or by the availability of macropores that provide low-resistance pathways for root growth in very strong soils (White and Kirkegaard, 2010). Root hairs have long been recognized as increasing the area of close root–soil contact, and so facilitating nutrient and water uptake by a plant (Farr, 1928; Nye and Tinker, 1977). The role of root hairs in soil penetration and in root anchorage is much less clear and few studies have explored this in detail, despite anchorage often being referred to as a function of root hairs (Grierson *et al.*, 2014; Wang *et al.*, 2014; Li and Lan, 2015). Interestingly, when the reference trail is followed back, two of these three references are unsupported by any citation on anchorage, whilst the other refers to a 1991 book review (Hofer, 1991) that, in turn, cites a much older review (Farr, 1928) which, in turn, cites an 1883 German text (Schwarz, 1883). The Schwarz (1883) paper appears to contain largely qualitative statements about the potential role of root hairs in anchorage, rather than a quantitative experimental study on anchorage. Bailey *et al.* (2002) measured the uprooting resistance of *Arabidopsis thaliana* wild type and hairless (*rdh2–1*) mutants. They concluded that root hairs did not contribute to the uprooting force for a whole *Arabidopsis thaliana* plant, but these authors did not test root hair anchorage of individual growing root tips where the geometry of the root–soil interaction is very different from that of a whole plant.

The role of root hairs in root tip anchorage was suggested by Stolzy and Barley (1968). When measuring the frictional force to withdrawing pea (*Pisum sativum* L.) radicles from soil they noted that the root appeared to become anchored in the soil by root hairs. The force required to withdraw a pea root increased to 0.1–0.2N mm^–1^ length of hairy root, much greater than the 0.04N mm^–1^ of root tip before root hair emergence. However, these results were not reported in detail, and seemed to be included to justify them choosing to measure roots before any root hairs had emerged. Information on soil–root hair adhesion emerged from a peel test developed by Czarnes *et al.* (1999). Adhesion was greatest for fine-textured soils, having a root–soil interfacial rupture energy of about 12 mJ m^−1^ for clay, 3–5 mJ m^−1^ for silty soil, and <1 mJ m^−1^ for sandy soil. Soil matric potential had, relatively, much less effect than texture, decreasing from 5 mJ m^−1^ to 3 mJ m^−1^ in silty soil as the matric potential decreased from −10 to −100 kPa.

Root hairs, theoretically, have sufficient strength to anchor a growing root tip to the surrounding soil. Estimates of the combined tensile strength of root hairs suggested that approximately 165 root hairs would be sufficient to anchor a maize root in moderately compacted soil if the entire strength of the root hairs is mobilized simultaneously (Bengough *et al.*, 2011). The practical importance of this for root penetration was demonstrated for barley roots growing from a loose (1.2g cm^−3^) surface layer of soil into a more compacted layer (1.7g cm^−3^; Haling *et al.*, 2013). Eighty-eight per cent of wild-type roots of barley penetrated the compacted soil layercompared with only 1% of the hairless barley mutant roots, indicating that, under certain circumstances, root hairs greatly facilitate soil penetration. A robotic system has been developed that mimics some selected physiological adaptations of plant roots for soil penetration (Sadeghi *et al.*, 2014; Lucarotti *et al.*, 2015). Penetration of granular soil by the robot was facilitated by anchoring the rear section of the robot to the surrounding matrix. This decreased the energy required for soil penetration by 50–75%, relative to a similar robot without this anchorage system.

Direct measurements are required to quantify root hair anchorage and to determine its importance for the penetration of root tips into soil of different strengths, as root hair elongation depends on the physical properties of the soil. Root hair length was shorter for barley roots grown in denser soil, being about 0.8mm in soil of a dry bulk density of 1.2g cm^−3^ compared with about 0.4mm for soil at 1.7g cm^−3^ (Haling *et al.*, 2014). Shorter root hairs will decrease the ability of root hairs to anchor the growing root tip in soil if an insufficient length of each root hair is secured within the rhizosphere soil pores. It is therefore likely that root hair anchorage will be optimal at intermediate soil densities.

In this paper, experiments were performed (a) to measure the anchorage force during pull-out of wild-type roots and hairless root mutants grown in artificial cylindrical biopores; (b) to determine whether root hairs influence the penetration of the root tip into soil from an artificial cylindrical biopore; and (c) to use time-lapse imaging to analyse root and soil displacements in both of these systems.

## Materials and methods

### Plant material

**Fig. 1.**
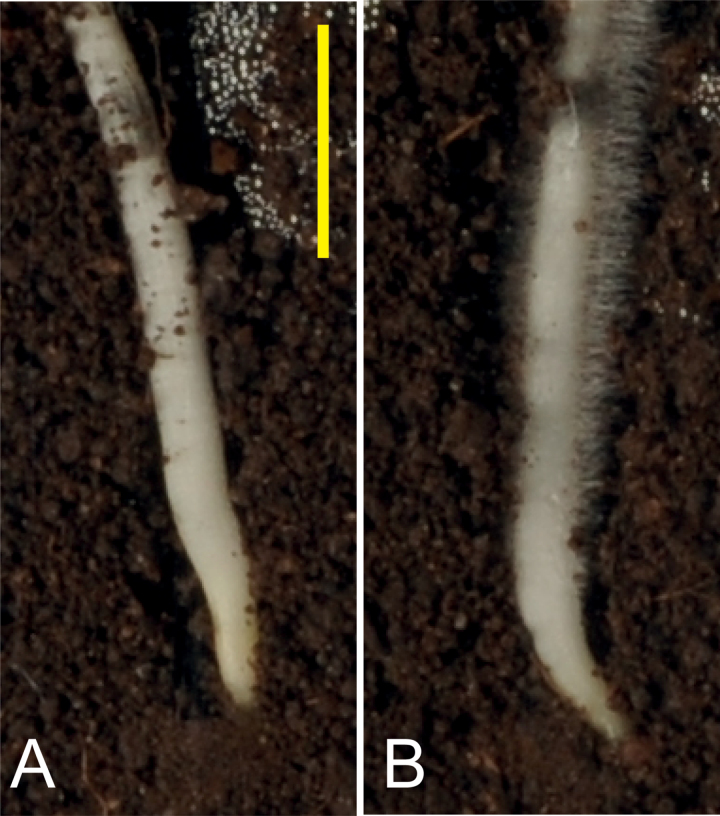
Wild-type (A) and hairless mutant (*rth3–3;* B) maize roots used in the experiments, growing from artificial bipore into soil of density 1.0g cm^−3^ (scale bar indicates 5mm).

Maize wild-type plus hairless mutant caryopses (Fig. 1) were surface-sterilized with 2% calcium hypochlorite solution, then rinsed five times in deionized water. Caryopses were left to germinate on damp blotting paper for 3 d at 20 ºC, until the primary root was between 10mm and 30mm long.

### Soil preparation

Sandy loam soil (mid-Pilmore field, from The James Hutton Institute, Dundee) was sieved <2mm, then rewet to 0.20g g^–1^ gravimetric water content (chosen to provide adequate soil water and aeration for root elongation); matric potential −38 to −61 kPa; Table 1), and left to equilibrate overnight in double-wrapped polythene bags. Soil was repacked into either (a) soil core rings or (b) soil boxes with transparent plastic (Perspex) sides. (a) Soil core rings (56mm diameter×40mm deep) were packed to dry bulk densities of 1.0, 1.2, 1.3, 1.4, or 1.5g cm^–3^. Soil core ends were sealed temporarily with polythene caps and double-wrapped polythene bags to equilibrate for 3 d. (b) Soil boxes (210 mm×230 mm×13mm) were packed to dry bulk densities of 1.0 or 1.2g cm^–3^. Soil boxes were double-wrapped in polythene bags to minimize moisture loss prior to the experiments.

**Table 1.** Penetrometer resistance, matric potential, and air-filled porosity (assumes density of solids, 2.65g cm^−3^) as a function of soil dry bulk density (mean values ±SEM, *n*=3 replicates)

Dry bulk density (g cm^−3^)	Penetrometer resistance (MPa)	Matric potential (–kPa)	Air-filled porosity (cm^3^ cm^−3^)
1.0	0.46±0.060	38±0.5	0.42
1.2	1.05±0.069	39±1.1	0.31
1.3	1.40±0.035	38±7.8	0.25
1.4	2.64±0.26	55±0.6	0.19
1.5	3.92±0.41	61±3.8	0.13

### Soil properties

Penetrometer resistance was measured using a penetrometer probe (1mm diameter, 1mm min^−1^ penetration rate, 30° cone angle, shaft diameter 0.8mm) driven into the soil by an Instron loading frame. Soil matric potential was measured using a 5mm diameter micro-tensiometer connected to a datalogger (SWT-5 UMS tensiometer, with DL6Te datalogger; both supplied by Delta-T devices, Cambridge, UK).

### Experiment 1a. Measuring anchorage force during the pull-out of roots from soil cores

Four holes were made vertically traversing each soil core, using a 2mm diameter drill bit. Seedlings were selected in random order and the initial root length was measured with a ruler. Seedlings were inserted gently into the hole in the soil core, such that the maize caryopsis sat on the soil surface. A second (empty) metal core ring was secured below the first with masking tape, covered with a layer of polythene, and filled with very loosely packed sieved soil. The root was left for approximately 2 d in an incubator at 20 ºC to grow through the hole and into the loose soil below.

**Fig. 2.**
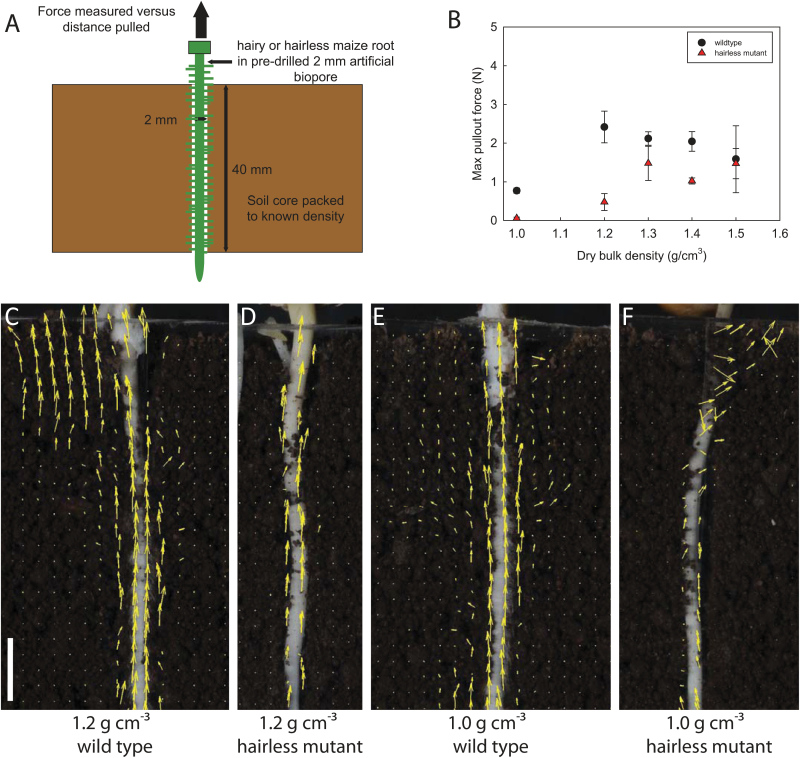
(A) Schematic diagram showing a maize root grown through an artificial cylindrical biopore in a soil core, prior to root pull-out. (B) Plot of maximum pull-out force as a function of soil dry bulk density. (C, D, E, F) Vector displacement fields measured from images during pull-out of roots grown in cylindrical biopores adjacent to a transparent plastic interface. The scale for panels C–F is the same and is indicated by a white bar in (C), representing 5mm. In all figures the yellow vectors are spaced on a regular grid and represent displacement, with the longest vector representing a displacement of 0.1mm (i.e. vectors are magnified relative to the scale of the underlying image). (C, E) Wild-type seedlings; (D, F) hairless mutants. (C, D) Soil dry bulk density 1.2g cm^−3^;(E, F) soil bulk density 1.0g cm^−3^.

At the end of the growth period, the loose soil was gently removed from the core below (Fig. 2A). The force required to pull the seedling from the core vertically at a rate of 1mm min^–1^ was measured with an Instron 5544 Universal loading frame. The base of the caryopsis was clipped to the base of the Instron load cell. The peak load, generally achieved at around 1.5–3mm displacement, was recorded for each seedling. Two replicate cores were used for each of the five soil bulk densities and there were two replicate seedlings for each genotype per core.

### Experiment 1b. Imaging root anchorage during the pull-out of roots grown into soil boxes

Four 10cm deep vertical holes were made in the soil box adjacent to the Perspex face using a rotating 2mm diameter drill bit to remove soil and to minimize fracturing. Hairless and mutant seedlings were selected in random order and inserted in the holes such that the maize caryopses sat on the soil surface. Seedlings were covered with polythene to prevent evaporation and left to grow for 2 d in an incubator at 20 ºC. At the end of the growth period the base of the caryopsis was clipped to the base of the Instron load cell, as in Experiment 1a. Two replicate soil boxes were imaged for 1.0 and 1.2g cm^−3^ soil bulk densities and there were two replicate seedlings for each genotype per core. Images were captured during root pull-out at 6s (equivalent to 0.1mm crosshead displacement) intervals using a Nikon D300 camera with macro lens (Nikkor AF-S MicroNikkor 60mm 1:2.8) and Nikon Pro time-lapse software.

### Experiment 2a. Root penetration into soil cores: seedling growth

**Fig. 3.**
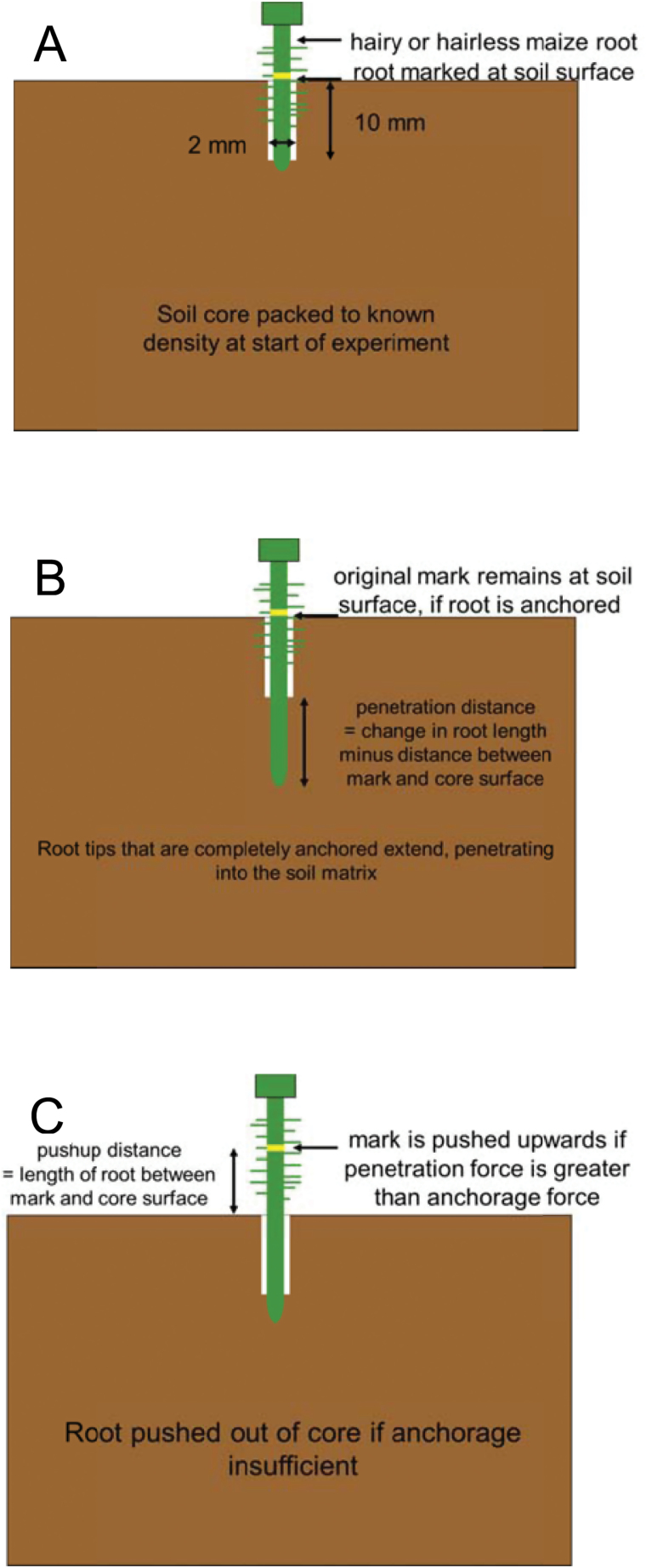
Schematic diagram (Experiment 2) showing the experimental set-up to study root anchorage during root growth from an artificial cylindrical biopore into bulk soil. (A) Initial setup. (B) Illustration showing a root that anchors itself in the biopore, enabling the root tip to penetrate further into the soil. (C) Illustration showing a root that does not anchor itself in the soil and so penetrates only a very short distance into the soil, with the majority of root extension directed out of the soil.

Two holes were made to a 10mm depth in the surface of each soil core using a 2 mmdiameter drill bit as in Experiment 1b. Seedlings were selected in random order and the initial root length was measured to ±0.5mm with a ruler. Seedlings were inserted gently into the hole in the soil core and the root was marked with ink at the level of the core surface (Fig. 3A). A second (empty) metal core ring was secured on top of the first with masking tape, covered with a layer of polythene. The root was left to grow for approximately 24h in an incubator at 20 ºC.

The length of the root above the surface of the core was recorded, together with the time, and the distance between the mark on the root and the soil surface (Fig. 3B, C). The primary root length of each seedling was then measured using a ruler after removal from the soil. The soil was then oven dried at 105 ºC to check the dry bulk density and water content.

### Experiment 2b. Imaging root penetration into soil boxes

Time-lapse imaging was performed to examine the root–soil physical interactions when roots were grown in the soil boxes adjacent to the transparent Perspex interface. Soil was packed to dry bulk densities of 1.0 or 1.2g cm^−3^ and at a gravimetric water content of 0.2g g^−1^. Maize seedlings were grown for a period of about 48h in 1cm deep preformed holes within the soil (made with a 2mm diameter drill bit, as in Experiment 2a). Boxes were tilted slightly forward to ensure that roots grew down the Perspex surface with the camera set up perpendicular to the Perspex surface. Images were captured at 15min intervals (with camera equipment described in Experiment 1b).

### Image analysis and statistics

Images were analysed using Particle Image Velocimetry (GeoPIV8 program, running in Matlab; White *et al.*, 2003) to quantify the displacement of root tissue and soil particles as a function of time in Experiment 1b. Root tip displacement coordinates were recorded manually as a function of time in Experiment 2b using ImageJ software (ImageJ, National Institutes for Health, USA). Image sequences were imported into ImageJ and the measurement facility was used to record point coordinates after selecting the root tip on the zoomed-in image in each image frame.

Where appropriate, arithmetic mean values were calculated for force, displacement, and growth-related parameters (values quoted are generally arithmetic mean ±standard error of the mean). T-tests were used to identify statistically significant differences between means. Fisher’s Exact Test was used to test whether the number of seedling roots that anchored themselves securely was significantly affected by the presence of root hairs.

## Results

### Soil properties

Penetrometer resistance increased from 0.5 to 4MPa with increasing soil dry bulk density, while air-filled porosity ranged from 0.13 to 0.42cm^3^ cm^−3^ (Table 1). Soil matric potential was between −38 and −61 kPa (Table 1), being more negative for the two densest soil treatments.

### Experiment 1a. Measuring anchorage force during the pull-out of roots from soil cores

The peak force required to pull the roots from the cores was typically achieved at 1.5–3.0mm displacement and is shown in Fig. 2B. Force for the wild type increased from 0.77±0.067N in the loosest soil, to a maximum of 2.4±0.41N in the 1.2g cm^−3^ density soil, above which it remained relatively constant. The mean maximum pull-out force for the hairless mutant was significantly smaller than the wild type for the 1.0, 1.2, and 1.4g cm^−3^ treatments, with this difference being greatest for the two loosest soils. The maximum pull out force was 13-fold greater for the wild type in the 1.0g cm^−3^ treatment (*P* <0.001) and 5-fold greater in the 1.2g cm^−3^ treatment, respectively, but no significant difference was found for the 1.3 and 1.5g cm^−3^ treatments (*P* >0.05). The pull-out force can be used to estimate the maximum reaction force (anchorage) that the root hairs provide to counter the force required for penetration of the root tip.

### Experiment 1b. Imaging root anchorage during the pull-out of roots grown into soil boxes

The wild-type roots disrupted a much greater region of soil around them during pullout (Fig. 2C, E, cf. D, F), thus mobilizing greater soil mechanical resistance than the hairless mutant roots. This is illustrated by the magnitude of the displacement vectors, indicated by arrows in Fig. 2C and, in particular, the horizontal distance from the root surface that substantial soil displacements are located: in the case of the wild type, displacements were found up to four times the root radius from the root surface, whereas the hairless mutant hardly disturbed the surrounding soil.

### Experiment 2a. Root penetration into soil cores: seedling growth

**Fig. 4.**
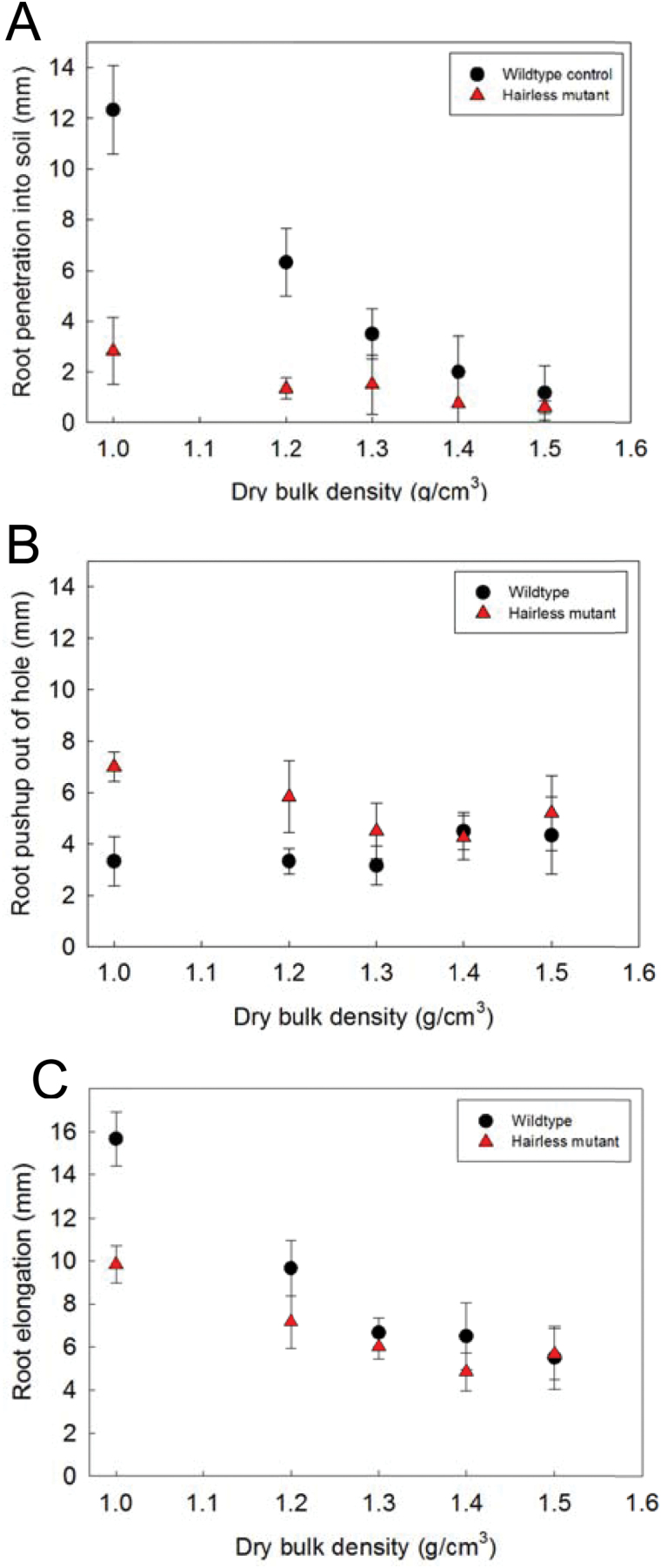
Root growth result (Experiment 2) showing (A) root tip displacement (penetration) into soil as a function of soil dry bulk density; (B) length of root pushed out of the artificial biopore as a function of soil dry bulk density; and (C) root elongation (increase in root length per unit time) as a function of soil dry bulk density.

Root penetration into the soil core was consistently deeper for the wild-type roots than for the hairless mutant roots (Fig. 4A): the difference was greatest for the 1.0 and 1.2g cm^−3^ treatments (*P* <0.01), becoming non-significant as densities increased from 1.3g cm^–3^ to 1.5g cm^−3^ (*P* >0.05). The length of root pushed up out of the hole was accordingly greater for the hairless mutant in the loosest soil (*P* <0.01 for 1.0g cm^−3^), becoming non-significant (*P* >0.05) as density increased to 1.2g cm^−3^ or greater (Fig. 4B). Total root elongation was greater for the wildtype than for the mutant roots in the loosest soil (Fig. 4C). This difference reflects a faster elongation rate in totally unimpeding conditions (e.g. unimpeded filter-paper-grown mutant roots elongated at70–75% of the rate of the wild-type roots). As the soil density increased. the better-anchored wild-type roots will have experienced greater mechanical impedance than the poorly-anchored mutant roots, causing this difference in elongation rates to diminish.

### Experiment 2b. Imaging root penetration into soil boxes

**Fig. 5.**
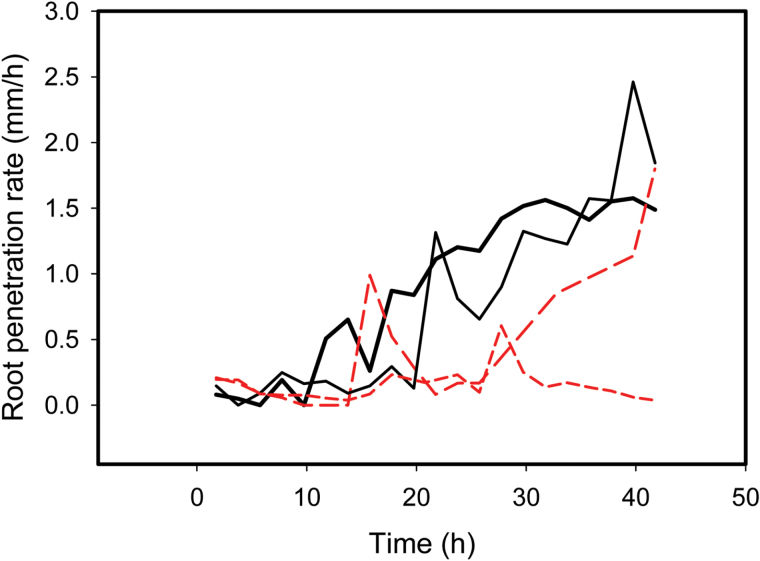
Rate of root penetration (root tip displacement per unit time) of four sample root tips as a function of time for wild-type (black solid lines; two replicates) and hairless mutant roots (red dashed lines; two replicates). Root tips penetrated the soil only when there was sufficient anchorage to provide the reaction force to counter the soil resistance to penetration. The two wild-type roots became anchored after about 11h and 21h. One mutant became anchored after about 26h while the remaining mutant became partially anchored at 13h, subsequently losing its anchorage grip. Roots were growing in a single soil box packed to dry bulk density of 1.2g cm^−3^.

The position of the root tip was tracked as function of time and used to estimate the penetration of the root tip into the soil (the penetration rate was calculated as the rate of root tip displacement per unit time; Fig. 5). In all cases, the growing root tissue was initially pushed vertically out of the soil unless the growing zone of the root became anchored (see Supplementary videos S1–S4 at *JXB* online). All of the wild-type roots became anchored within 38h, with the mean anchorage time being 25h in the 1.0g cm^−3^ soil and 16h in the 1.2g cm^−3^ soil (Table 2; see examples in Fig. 5). By contrast, four out of 10 hairless roots never became anchored in the soil, with a further four roots only becoming partially anchored (such that root tissue was displaced in both directions – out of the soil and into the soil – as the elongating root tissue extended). The number of seedling roots that anchored themselves securely was significantly increased by the presence of root hairs (*P* <0.001). The time that the hairless roots grew in the 1.2g cm^−3^ soil with no anchorage was significantly longer than that for the wild-type roots (33.3h cf. 16.3h; Table 2; *P* <0.05: e.g. Fig. 5). The distance that each root tip penetrated, relative to the surrounding soil, before it was judged to be anchored was <3% of the total root tip displacement for each root tip. Penetration rate varied substantially as a function of time between individual roots, presumably depending on the small-scale physical interactions between the root surface and the biopore wall.

**Table 2.** Time until roots anchored themselves Values are mean ±SEM (*n*=4 replicates for 1.0g cm^−3^; *n*=6 for 1.2g cm^−3^).

Dry bulk density (g cm^−3^)	Time to anchorage (h)	
	Hairless mutant	Wild type
1.0	35.0±7.5	25.0±4.8
1.2	33.3±5.4	16.3±3.4

## Discussion

### Soil physical properties

Soil physical properties were chosen such that mechanical impedance was likely to be the major limitation to root elongation in the denser soil cores (Table 1; Bengough *et al.*, 2011). Air-filled porosity was greater than 0.13cm^3^ cm^−3^ for all treatments, suggesting that hypoxia was unlikely to be a limit to root elongation. Similarly, matric suctions <70 kPa should not significantly limit root elongation via a restriction on water supply. Penetrometer resistance of 2MPa is often taken as an indicator of where mechanical impedance will be a major limitation to root growth (Bengough et al 2011) and this was exceeded for the two densest treatments, with a consistent pattern of increasing penetrometer resistance with increasing bulk density.

### Root tip anchorage increased with the presence of root hairs and enabled soil penetration

**Fig. 6.**
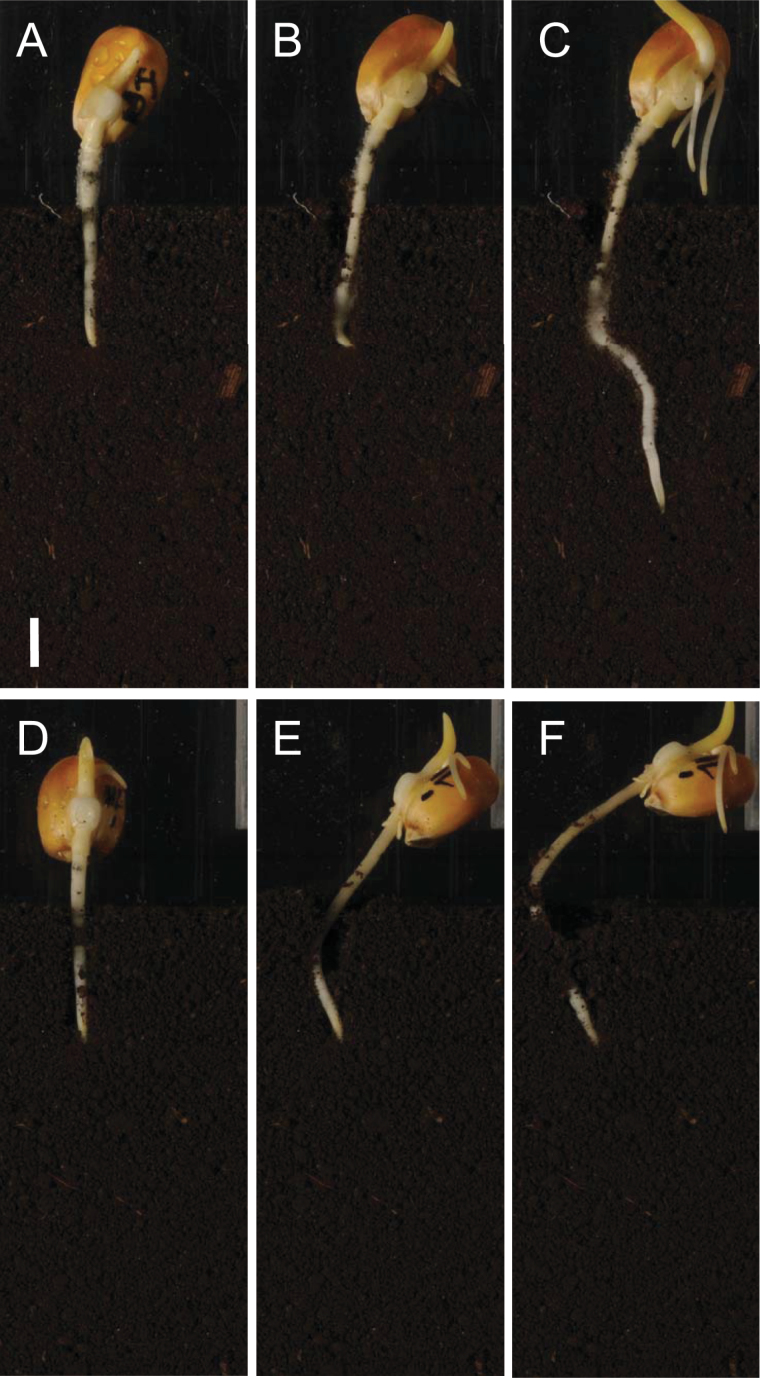
Image sequence showing two maize seedlings (wild type, A, B, C; hairless mutant *rth3–3*, D, E, F) at 1h (A, D), 10h (B, E), and 43h (C, F) after insertion into biopores in soil in Experiment 2b. The primary root of both seedlings initially push the caryopsis out of the soil until the wild-type root anchors itself with root hairs (B) after about 10h. The root with hairs (B) then penetrates the soil. The hairless mutant never anchors itself securely in the soil, so the root tip does not penetrate significantly (E, F).

Wild-type seedlings had 5–13-fold greater pull-out resistances than hairless seedlings, with a maximum pull-out force of 2.4N for the 1.2g cm^−3^ treatment. Given that the strength of an individual root hair is likely to be around 0.0024N (Bengough *et al.*, 2011) this would correspond to the tensile strength of 1 000 root hairs being mobilized simultaneously. This suggests that root hairs can provide most of the anchorage force required for root penetration, resulting in much greater root tip penetration and much bigger soil displacements in the rhizosphere for wild-type than for mutant roots (Figs 2, 6). The maximum reaction force recorded during root penetration of pea roots in literature studies is typically around 1.8N (Clark *et al.*, 1999), which would be provided by a 3cm length of root covered with root hairs anchored in the 2mm biopore on the basis of our results here.

It is possible to calculate whether the anchorage provided by root hairs is of the same order of magnitude as that estimated theoretically in Bengough *et al.* (2011). The length of root axis pulled from the soil in Experiment 1a was 40mm. If there were 100 root hairs mm^−1^ of root axis (a reasonable estimate from the root in Fig. 1B) that would give 4 000 root hairs, and a maximum pull-out force of 4 000×0.0024 N× cos45°=6.8N if the strength of each root hair was mobilized when the hair was oriented at 45° to the direction of pull-out. Given that the individual degree of anchorage of many root hairs will be imperfect and that the maximum strength of all the root hairs will not be mobilized simultaneously, it appears that the estimates of root hair anchorage forces are well within an order of magnitude agreement with those made previously (Bengough *et al.*, 2011).

Root hairs enabled growing root tips to anchor themselves in the soil much more rapidly than the hairless mutants at soil densities of 1.0 and 1.2g cm^−3^, with the majority of the hairless roots never becoming securely anchored. Roots became anchored much faster in the 1.2g cm^−3^ soil than in the 1.0g cm^−3^ soil, probably due to the anchorage force increasing more rapidly with soil density than the soil penetration resistance (a factor of three for anchorage force, compared with a factor of two for soil penetration resistance; Table 1). The penetrometer resistance in the 1.2g cm^−3^ soil was 1.05MPa, which corresponds to a force of approximately 0.8N on a 1mm diameter penetrometer probe. Given that roots encounter only one-half to one-eighth of the soil penetration resistance experienced by a penetrometer probe (so, in this case perhaps around 0.2N; Bengough and Mullins, 1991), it is apparent that the strength of root anchorage provided by the root hairs (=2.4N/40 mm=0.06N mm^−1^) should be sufficient to allow root penetration if more than about 3–4mm of root is firmly attached to the soil by root hairs. It is equally possible to see that, in the 1.5g cm^−3^ soil, a 4-fold increase in penetrometer resistance (to approximately 4MPa) is combined with a weaker anchorage force (to approximately 1.6N/40 mm=0.04N mm^−1^), making penetration of the soil unachievable with the anchorage force that can be mobilized unless 25mm or more of root can be firmly attached to the soil. In this discussion it must be remembered that we are considering the situation of a root penetrating from a vertical biopore into bulk soil and that, if the entire root is very closely surrounded by dense soil or if the root trajectory twists substantially, there will probably be more than enough anchorage force available for soil penetration by the root tip. In a field situation the seed would, of course, be planted below the soil surface, increasing the normal load on the root axis and aiding root penetration of the young seedling.

Location of root hair emergence and anchorage varied greatly between replicate seedlings, and may depend on the micro-environment of the rhizosphere immediately adjacent to the root surface. After placing the wild-type roots in the artificial biopore in Experiment 2b, root elongation continued for a period of several hours before root hairs emerged and grew towards and into the biopore walls (Supplementary videos S1 and S2). In one example (Supplementary video S1), root hairs emerge relatively rapidly in the region 2–6mm from the root tip whilst, in another replicate seedling (Supplemenmtary video S2), root hairs are slower to emerge, but do so along a much greater length behind the root apex. In both cases, anchorage is achieved fairly rapidly, but it is not clear why root hair formation differs substantially between these replicates. For wheat roots growing in dense structured subsoil, the density of root hairs (number per mm of root) decreased approximately exponentially with increasing contact with the pore walls (White and Kirkegaard, 2010). This suggests that the degree of close contact may influence root hair emergence in any given location. Hirth *et al.* (2005) found that walls of more horizontally oriented biopores were penetrated by ryegrass root tips more rapidly than vertically oriented biopore walls and that pore walls with rougher surfaces were penetrated by more successfully than smooth-walled pores. In the experiments performed within the present study, the pore walls would be relatively rough as the pores were excavated using a drill bit.

The mass of rhizosheath associated with barley seminal roots varies greatly according to plant genotype (George *et al.*, 2014). The mass of rhizosheath soil adhering to young seminal roots varied more than 5-fold between elite spring barley cultivars, and by more than 12-fold between mutant phenotypes, with part of this variation being explained by root hair length. This suggests that different crop cultivars may have varying degrees of success in exploiting biopores and cracks in structured soils. Indeed, the pattern of root growth within biopores has been found to depend markedly on plant species (Athmann *et al.*, 2013): Although a similar fraction (85%) of both barley and oilseed rape roots contacted the pore walls of biopores in horizons of silty loam or silty clay loam, the pattern of root growth within the biopores differed markedly between these species. Barley main root axes contacted the pore walls closely, often following the pore with a helical pattern of growth. By contrast, main root axes of oilseed rape often grew more centrally down the pore, relying on lateral roots to contact the pore walls.

In conclusion, root hairs provide anchorage for individual maize root tips growing in soil pores and this anchorage can aid root penetration. Root hairs enable roots to fix themselves to soil pore walls and may enable the penetration of main or lateral axes into the bulk soil matrix surrounding a biopore. The degree of anchorage provided by root hairs will depend substantially on both phenotype (root hair and possibly mucilage production) and on the microstructure of the rhizosphere (that will determine the depth to which root hairs can penetrate within and adhere to the soil matrix).

## Supplementary data

Supplementary data can be found at *JXB* online.

Video S1. Timelapse video in Experiment 2b for a wildtype maize root growing in a soil box packed to 1.2g cm^-3^. The root quickly became anchored by root hairs and penetrated the soil successfully (frame rate is replayed at 30 frames/s with the original timelapse images taken at 15min intervals).

Video S2. Timelapse video in Experiment 2b for a replicate wildtype maize root growing in a soil box packed to 1.2g cm^-3^. Relatively quickly the root again became anchored by root hairs and penetrated the soil successfully (frame rate is replayed at 30 frames/s with the original timelapse images taken at 15min intervals).

Video S3. Timelapse video in Experiment 2b for a hairless (*rth3-3*) mutant maize root growing in a soil box packed to 1.2g cm^-3^. The root never became securely anchored, such that root elongation simply pushed the seedling out of the soil, instead of helping the root tip to penetrate further (frame rate is replayed at 30 frames/s with the original timelapse images taken at 15min intervals).

Video S4. Timelapse video in Experiment 2b for a replicate hairless (*rth3-3*) mutant maize root growing in a soil box packed to 1.2g cm^-3^. At first the root was not securely anchored, and so pushed out of the soil. After many hours the root tip became able to penetrate the soil, probably due to the bend in the root providing sufficient reaction force to overcome the resistance to penetration (frame rate is replayed at 30 frames/s with the original timelapse images taken at 15min intervals).

Supplementary Data
