# Supplementary material for: Root hairs aid soil penetration by anchoring the root surface to pore walls
Source: J Exp Bot. 2016 Jan 21;67(4):1071–8. doi: 10.1093/jxb/erv560 (PMC4753853; doi:10.1093/jxb/erv560)
Supplement: Supplementary Data [file supp_67_4_1071__index.html]

Root hairs aid soil penetration by anchoring the root surface to pore walls — Root hairs aid soil penetration by anchoring the root surface to pore walls — Supplementary Data 

# Root hairs aid soil penetration by anchoring the root surface to pore walls

## Supplementary Data

Data files

- supplementary\_video\_S1.gif - Supplementary Data
- supplementary\_video\_S2.gif - Supplementary Data
- supplementary\_video\_S3.gif - Supplementary Data
- supplementary\_video\_S4.gif - Supplementary Data
