# Supplementary figures and images for: Root hairs aid soil penetration by anchoring the root surface to pore walls
Source: J Exp Bot. 2016 Jan 21;67(4):1071–8. doi: 10.1093/jxb/erv560 (PMC4753853; doi:10.1093/jxb/erv560)

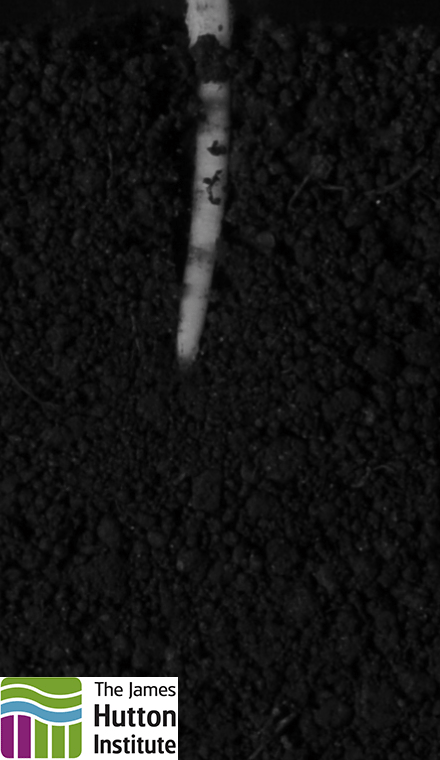

Supplement: Supplementary Data [file supp_erv560_supplementary_video_S1.gif]

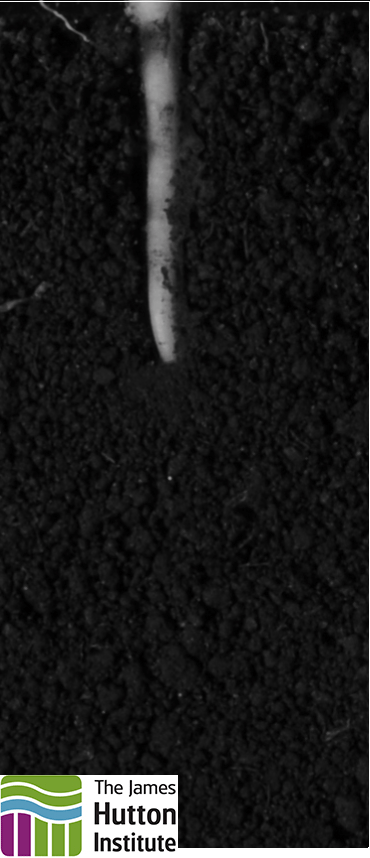

Supplement: Supplementary Data [file supp_erv560_supplementary_video_S2.gif]

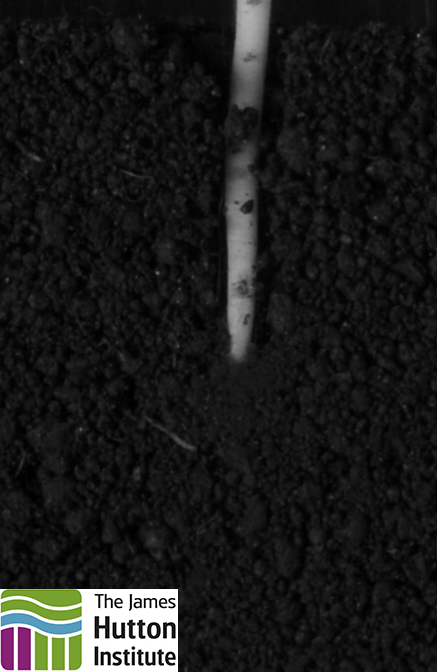

Supplement: Supplementary Data [file supp_erv560_supplementary_video_S3.gif]

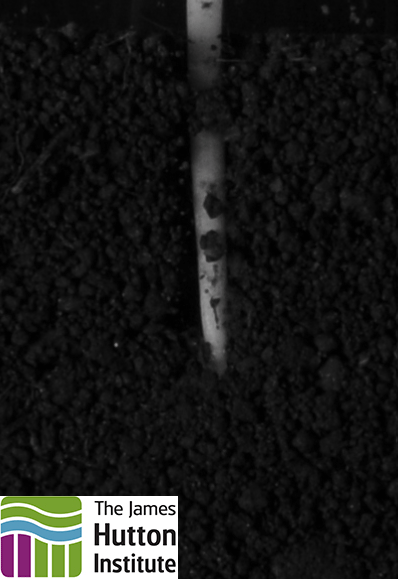

Supplement: Supplementary Data [file supp_erv560_supplementary_video_S4.gif]
